# Fucoidan-Mediated Inhibition of Fibrotic Properties in Oral Submucous Fibrosis via the MEG3/miR-181a/Egr1 Axis

**DOI:** 10.3390/ph15070833

**Published:** 2022-07-05

**Authors:** Chih-Yuan Fang, Szu-Han Chen, Chun-Chung Huang, Yi-Wen Liao, Shih-Chi Chao, Cheng-Chia Yu

**Affiliations:** 1 School of Dentistry, College of Oral Medicine, Taipei Medical University, Taipei 110, Taiwan; 100044@w.tmu.edu.tw; 2 Division of Oral and Maxillofacial Surgery, Department of Dentistry, Wan Fang Hospital, Taipei 116, Taiwan; 3 School of Dentistry, Chung Shan Medical University, Taichung 40201, Taiwan; jasminne1117@gmail.com; 4 Institute of Oral Sciences, Chung Shan Medical University, Taichung 40201, Taiwan; seanabo@gmail.com; 5 Department of Medical Research, Chung Shan Medical University Hospital, Taichung 40201, Taiwan; rabbity0225@gmail.com; 6 Department of Medical Research and Education, Lo-Hsu Medical Foundation, Lotung Poh-Ai Hospital, Yilan 265501, Taiwan; 7 Department of Dentistry, Chung Shan Medical University Hospital, Taichung 40201, Taiwan

**Keywords:** oral submucous fibrosis, MEG3, miR-181a, Egr1, myofibroblasts, competing endogenous RNA

## Abstract

Oral submucous fibrosis (OSF) is a chronic fibrotic remodeling disease that can progress to oral cancer. However, efficient clinical diagnosis and treatment methods for OSF are still lacking. This study investigated the anti-fibrotic effect of fucoidan on oral fibrosis. To evaluate the fibrotic ability (myofibroblast activities), we performed wound-healing, Transwell migration, and collagen contraction assays by using patient-derived normal and fibrotic buccal submucous fibroblasts (BMFs and fBMFs, respectively). RNA-sequencing and dual-luciferase reporter and RNA immunoprecipitation chip assays were performed to identify the clinical significance and molecular mechanism of non-coding RNAs. Fucoidan suppressed the myofibroblast activities and inhibited the MEG3 in fBMFs. MEG3 was overexpressed in the OSF tissue and was positively associated with myofibroblast markers. Knockdown of MEG3 markedly inhibited myofibroblast activities, which were restored by inhibiting miR-181a and overexpressing Egr1. The results from luciferase reporter and RIP assays confirmed that MEG3 functioned as a competing endogenous RNA (ceRNA) and could directly target miR-181a, thereby preventing the miR-181a-mediated translational repression of Egr1. This study demonstrated that MEG3 exerts a profibrotic effect on OSF by targeting miR-181a/Egr1. Therefore, the administration of fucoidan may serve as a potential therapeutic strategy for OSF by targeting the overexpression of MEG3.

## 1. Introduction

Oral submucous fibrosis (OSF) is an irreversible fibrotic disease that frequently occurs at the oral buccal mucosa and is caused by areca nut chewing. The deposition of the fibrotic tissue results in restricted mouth opening, which leads to various problems, including difficulty in eating, phonation, oral hygiene maintenance, and malignant lesion screening. OSF is regarded as a precancerous state, and the malignant transformation rate ranges from 7.6% to 8.6% [1,2,3,4,5]. The prognosis of patients with advanced oral cancer is poor [6], and difficulty in mouth opening may hinder these patients from undergoing oral examination. Despite advances in cancer treatment, the current modalities available for treating premalignant OSF are not satisfactory. To prevent the progression of OSF into oral cancer, gaining a better understanding of the molecular mechanisms underlying the pathogenesis of OSF is crucial.

Various studies have reported that the constituents of areca nut are responsible for the development of OSF [2,7,8,9]. Van der Bijl et al. revealed a significant diffusion level of arecoline, the main areca nut alkaloid, by using both human vaginal and oral buccal mucosa specimens in in vitro experimental models [10,11]. Wen et al. developed a mouse model of arecoline-induced oral mucosal fibrosis. They reported that the OSF-like changes in mucosal epithelium, such as gradual flattening and atrophy, were observed in the early stage. Furthermore, abnormal collagen deposition and decreased density of blood vessels occurred in the connective tissue over time [12]. These could probably explain why exposure to arecoline causes epithelium atrophy, which is regarded as a common characteristic in the human OSF tissue. These architecture changes may alter the permeability of the mucosa, leading to the loss of barrier function and allowing a higher penetration level of arecoline that further consolidates the connective tissue fibrosis [13,14].

Fibrosis is primarily mediated by fibroblasts, which can transdifferentiate into myofibroblasts and disturb the extracellular matrix (ECM) by excessive secreting of the ECM protein and associated cytokines and proteases. The persistent activation of myofibroblasts results in the dysregulation of ECM synthesis and degradation. [15,16]. Various studies reported that arecoline stimulates cells to secrete growth factors and cytokines that enhance collagen accumulation and reduce collagen degradation [17,18,19]. The transforming growth factor beta (TGF-β) signaling pathway is a major driver of the progression of epithelial to mesenchymal transition (EMT) [20]. Notably, arecoline-stimulated expression of TGF-β contributes to the development of OSF [21,22,23]. Moreover, Slug, an EMT transcriptional factor, mediates myofibroblast differentiation in OSF caused by areca-nut chewing [24]. However, the detailed mechanism underlying the regulation of myofibroblast activation remains to be elucidated.

The loss of long non-coding RNA (lncRNA) maternally expressed 3 (MEG3) expression is observed in various types of disorders, such as cancers [25,26] and fibrotic disease [27]. For instance, the expression of MEG3 was observed to be downregulated in TGF-β1-induced renal fibrosis [28]. Xue et al. reported that the overexpression of MEG3 reduced TGF-β1-induced EMT, cell viability, and proliferation [28]. However, MEG3 may play a pro-fibrosis role because it has been demonstrated to enhance fibrosis and inflammatory responses in diabetic nephropathy through the miR-181a/Egr1/TLR4 axis [29]. Therefore, investigating the effects of MEG3 on the progression of OSF may be advantageous to understand the underlying pathogenesis mechanisms.

Fucoidan is a marine sulfated polysaccharides present in brown seaweed and exhibits several biological activities, such as immunomodulatory [30,31] and antiangiogenic effects [32]. A clinical study investigated changes in miRNAs after the ingestion of fucoidan by examining blood samples and observed that 53 out of 754 miRNAs were affected by fucoidan [33]. Many lncRNAs were responsive to the fucoidan administration. For instance, the expression of several lncRNAs was altered following 48 h treatment with oligo-fucoidan in hepatoma cells, such as NEAT1, and MEG3 [34]. Moreover, fucoidan has been demonstrated to inhibit the renal tubulointerstitial fibrosis induced by transient ischemic injury [35], lung fibrosis induced by radiation [36] or bleomycin [37], and diabetes-associated renal fibrosis [38]. Various studies have indicated that the anti-fibrotic function of fucoidan is mediated by the TGF-β signaling pathway [35] and its targeting genes related to the EMT [37,39,40] and fibrogenic processes [38].

Given that numerous studies have demonstrated the anti-fibrotic effects of fucoidan, we hypothesized that fucoidan would attenuate the progression of OSF. Moreover, previous studies have suggested that fucoidan exerts its biological effects by modulating non-coding RNAs. Therefore, this study investigated the effect of fucoidan on fibrotic properties (myofibroblast activities) in patient-derived fibrotic buccal mucosa fibroblasts (fBMFs) and whether MEG3 is the most downregulated lncRNA following fucoidan administration. In addition, we verified our findings in clinical specimens and examined whether the silencing of MEG3 affects myofibroblast activities to identify potential therapeutic targets for OSF.

## 2. Results

### 2.1. Fucoidan Inhibits the Myofibroblast Activities of Fibrotic BMFs (fBMFs)

**Figure 1:**
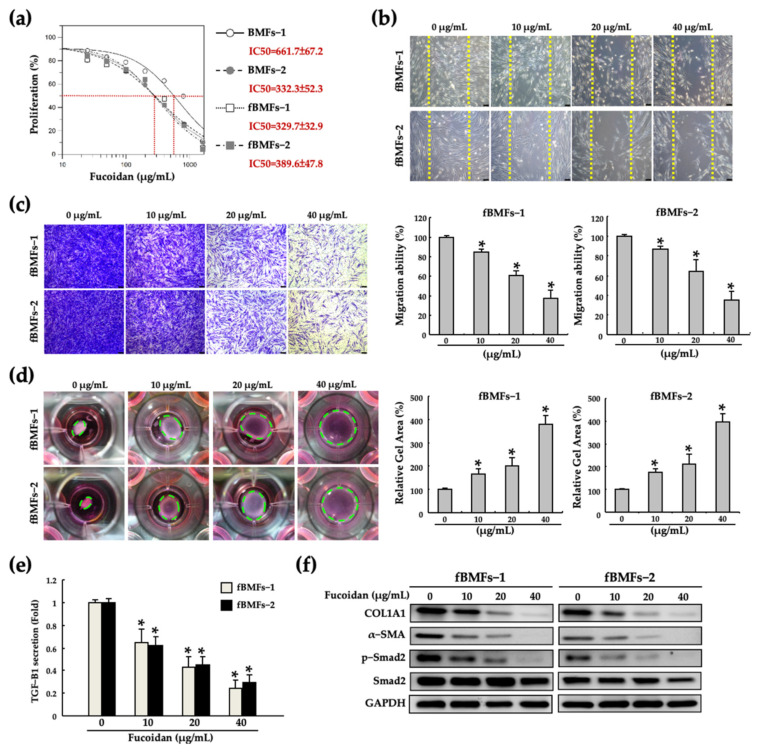
Fucoidan inhibited the myofibroblast features of patient-derived fBMFs without causing damage to normal BMFs. The effect of fucoidan on (**a**) the viability of both patient-derived BMFs and fBMFs; (**b**) wound healing ability, (**c**) Transwell migration, (**d**) gel contraction, (**e**) the TGF-β1 secretion and (**f**) myofibroblast related protein expression (COL1A1, α-SMA, p-Smad2 and Smad2) were examined in two individual fBMFs. * *p* < 0.05 compared with 0 (μg/mL).

To confirm the safety of fucoidan, we first examined the viability of fBMFs following treatment with fucoidan. As presented in Figure 1a, no significant difference in viability was observed between patient-derived normal BMFs and fBMFs after treatment with fucoidan in a series of concentrations. These results indicate that the cell viability was maintained above 80% when the concentration of fucoidan was lower than 50 μg/mL. Therefore, we chose 10, 20 and 40 μg/mL of fucoidan for the following experiments. Transformed myofibroblasts are responsible for the contraction of connective tissues to accelerate wound healing. However, myofibroblasts continuously exist and are persistently activated in the injured tissue, leading to fibrosis [41]. Thus, we performed wound healing, Transwell migration and collagen contraction assays to examine the anti-fibrotic effect of fucoidan on the myofibroblast activities of fBMFs. We observed that fucoidan significantly diminished wound healing (Figure 1b), migration (Figure 1c), and collagen gel contraction (Figure 1d) abilities. Moreover, fucoidan significantly downregulated myofibroblasts biomarkers, including the secretion of TGF-β1 (Figure 1e) and the expression of COL1A1, α-SMA, and p-Smad2 (Figure 1f). These findings indicate that a lower fucoidan concentration is adequate to suppress the myofibroblast features of fBMFs without damaging normal cells (BMFs).

### 2.2. Fucoidan Suppresses the Myofibroblast Activities of fBMFs by Reducing the MEG3 Levels

**Figure 2:**
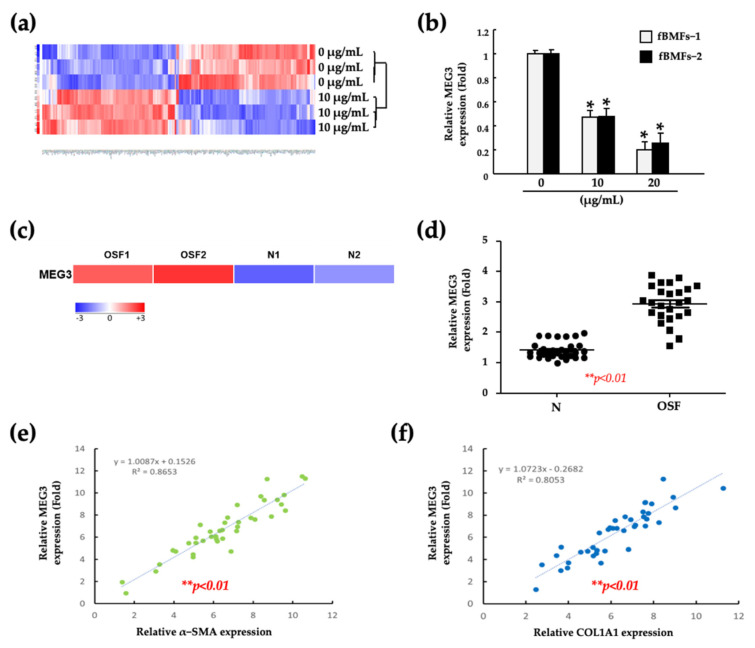
MEG3 was positively correlated with OSF. (**a**) The significantly differential expression of the lncRNA transcriptome in three individual patient-derived fBMFs after fucoidan treatments. (**b**) Changes in MEG3 expression in two individual patient-derived fBMFs in response to the indicated concentration of fucoidan. * *p* < 0.05 compared with 0 (μg/mL). (**c**,**d**) The expression of MEG3 in normal or OSF clinical specimens (*n* = 30); analysis of the relationship between MEG3 and two OSF-associated factors, namely (**e**) α-SMA and (**f**) COL1A1, by using oral cancer data from TCGA.

To elucidate the molecular mechanism underlying the inhibition of myofibroblast activities by fucoidan, we performed an RNA-sequencing analysis of fBMFs after treatment with 10 μg/mL fucoidan. As presented in Figure 2a, a heatmap of RNA-sequencing results revealed that MEG3 was among the most downregulated RNA by fucoidan. Moreover, the expression of MEG3 was decreased significantly in a dose-dependent manner following treatment with 10 and 20 μg/mL fucoidan (Figure 2b). To examine the role of MEG3 in oral fibrosis development, we detected the MEG3 level in paired normal and fibrotic specimens obtained from patients with OSF by performing RNA-sequencing and real-time quantitative polymerase chain reaction (qRT-PCR), respectively. The expression of MEG3 was significantly increased in the OSF specimen (Figure 2c,d). Furthermore, using oral cancer data from The Cancer Genome Atlas (TCGA), we observed that MEG3 was positively correlated with α-SMA and COL1A1 (Figure 2e,f). These results indicated that MEG3 can serve as a positive marker for OSF progression and a target for fucoidan treatment.

### 2.3. Silencing of MEG3 in fBMFs Suppresses the Myofibroblast Activities

**Figure 3:**
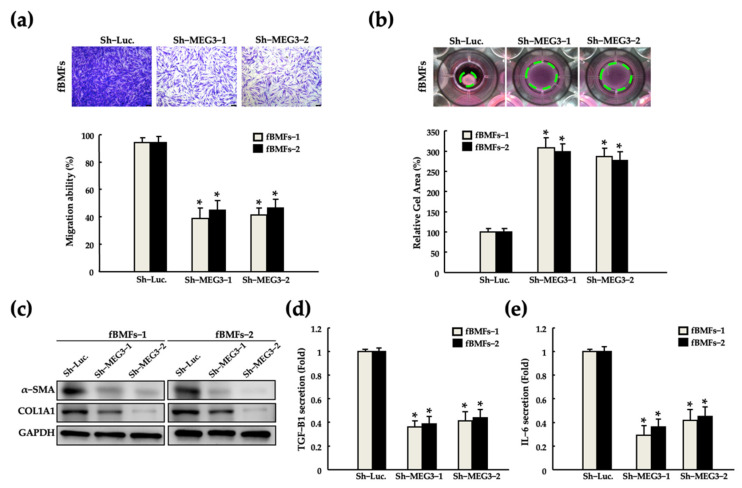
Silencing of MEG3 suppressed the myofibroblast features of patient-derived fBMFs. Changes in the (**a**) migration and (**b**) gel contraction abilities, (**c**) and expression of α-SMA and COL1A1, and the secretion of (**d**) TGF-β1 and (**e**) IL-6 was examined in two fBMFs after transfection with Sh-Luc. or Sh-MEG3. * *p* < 0.05 compared with Sh-Luc.

To verify the significance of MEG3 in the maintenance of myofibroblast activities, we silenced MEG3 in fBMFs by using a short-hairpin RNA (shRNA). Silencing of MEG3 significantly reduced the myofibroblast activities, including migration and collagen gel contraction abilities and a-SMA and COL1A1 expression (Figure 3a–c, respectively). Myofibroblasts have been reported to promote tissue inflammation by stimulating interleukin (IL)-6 secretion, thus leading to fibrosis and cancer progression [42,43,44,45]. Silencing of MEG3 suppressed the secretion of TGF-β1 and IL-6 from both fBMFs (Figure 3d). These findings suggest that the modulation of MEG3 expression may be a promising strategy to diminish the aberrant activation of fBMFs.

### 2.4. MEG3 Acts as a miR-181a Sponge in fBMFs

**Figure 4:**
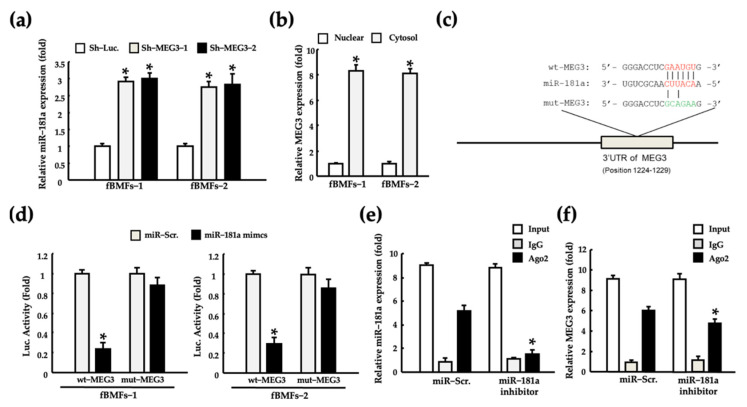
MEG3 inhibited the expression of miR-181a by acting as a miRNA sponge. (**a**) Changes in miR-181a expression in two patient-derived fBMFs after transfection with Sh-Luc or Sh-MEG3. * *p* < 0.05 compared with Sh-Luc. (**b**) The expression level of MEG3 in the cytoplasm and nucleus of two fBMFs analyzed by qRT-PCR. * *p* < 0.05 compared with the nucleus. (**c**) Putative base pairing sequence of wild-type (wt) and mutated (mut)-MEG3 with miR-181a. (**d**) Relative luciferase activity of each combination was examined in two fBMFs * *p* < 0.05 compared with miR-181a mimics. Relative expression of (**e**) miR-181a and (**f**) MEG3 in RNA immunoprecipitation chip (RIP) assay experiments was examined in two fBMFs after transfection with miR-Src or miR-181a inhibitor. * *p* < 0.05 compared with miR-Scr.

MEG3 functions as a miR-181a sponge to block the miR-181a-mediated inhibition of Erg1 mRNA translation, which results in diabetic kidney inflammation and fibrosis [46,47]. However, the exact molecular mechanism through which MEG3 leads to OSF progression remains unknown. The significant increase in miR-181a expression observed after MEG3 silencing suggests that MEG3 acts as a sponge of miR-181a (Figure 4a). On the basis of the understanding that competition interactions among lncRNAs occur in the cytoplasm, we first determined the subcellular location of MEG3 in fBMFs. MEG3 was expressed in the nucleus and cytoplasm of fBMFs and primarily localized in the cytoplasm (Figure 4b). To confirm whether MEG3 directly targets miR-181a, we used the miRDB bioinformatic database to identify the putative miR-181a targeting site on the 3’UTR region of MEG3 (Figure 4c). Luciferase reporter activity and RNA immunoprecipitation chip (RIP) assays were performed to determine the exact pairing of MEG3 to miR-181a. An miR-181a mimic significantly reduced the luciferase reporter activity in fBMFs cotransfected with wild-type (wt)-MEG3; however, luciferase reporter activity was considerably restored by con-transfected with mutated (mut)-MEG3 (Figure 4d). Furthermore, the miR-181a inhibitor markedly reduced miR-181a expression in Ago2 pellets (Figure 4e). This result indicates that miR-181a functions by forming the RNA-induced silencing complex (RISC) in fBMFs. In addition, we noted that the MEG3 expression was significantly reduced by the miR-181a inhibitor (Figure 4f). These findings suggest that MEG3 acts as a sponge for miR-181a in an RISC-dependent manner.

### 2.5. MEG3/miR-181a Axis Alters Myofibroblast Activites of fBMFs

**Figure 5:**
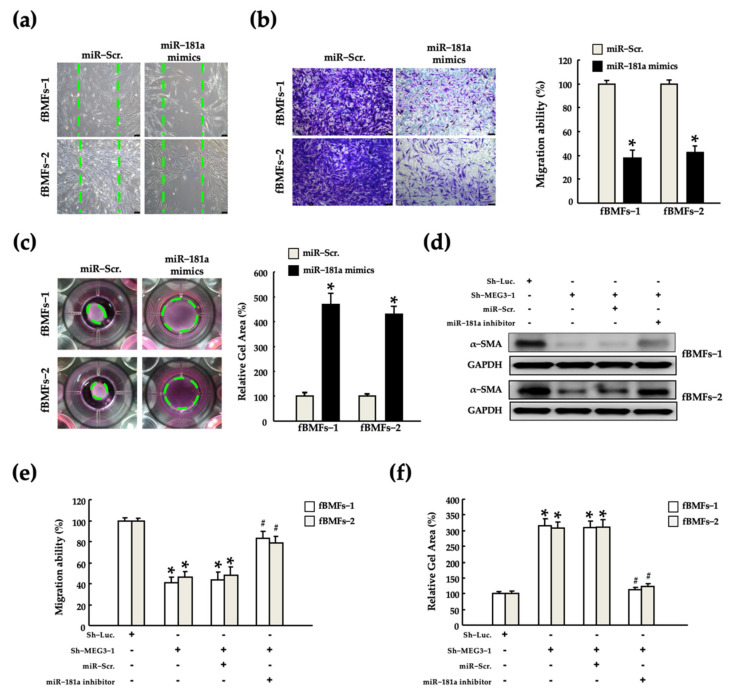
MEG3/miR-181a axis regulated myofibroblast activities in patient-derived fBMFs. Changes in (**a**) wound-healing, (**b**) migration, and (**c**) gel contraction abilities were examined in two fBMFs after transfection with miR-Scr or miR-181a mimics. * *p* < 0.05 compared with miR-Scr. Changes in (**d**) α-SMA expression and (**e**) migration and (**f**) gel contraction abilities were examined in two fBMFs after co-transfection with or without Sh-Luc, Sh-MEG3, miR-Scr, and miR-181a inhibitor, respectively. * *p* < 0.05 compared with Sh-Luc; # *p* < 0.05 compared with co-transfection with Sh-MEG3 and miR-Scr.

Our results revealed a strong positive correlation between MEG3 and myofibroblast activities (Figure 2 and Figure 3). Moreover, we noted that miR-181a exerted an antagonistic effect on MEG3 (Figure 4). Thus, we investigated the effects of miR-181a on the myofibroblast activities by performing wound healing, Transwell migration and gel contraction assays in fBMFs after transfection with miR-181a mimics to confirm whether miR-181a acts as an anti-fibrotic miRNA. We noted that miR-181a suppressed the migration and gel contraction abilities significantly (Figure 5a–c). Furthermore, we examined whether MEG3 stimulates myofibroblast activities by inhibiting miR-181a. Silencing of MEG3 markedly reduced the expression of α-SMA expression, and the abilities of migration and gel contraction (Figure 5d–f). This inhibitory effect was partially reversed following cotransfection with a miR-181a inhibitor. These findings suggest that MEG3 exerts a pro-fibrotic effect by inhibiting miR-181a.

### 2.6. MEG3/miR-181a/Erg1 Axis Alters the Myofibroblast Activities of fBMFs

**Figure 6:**
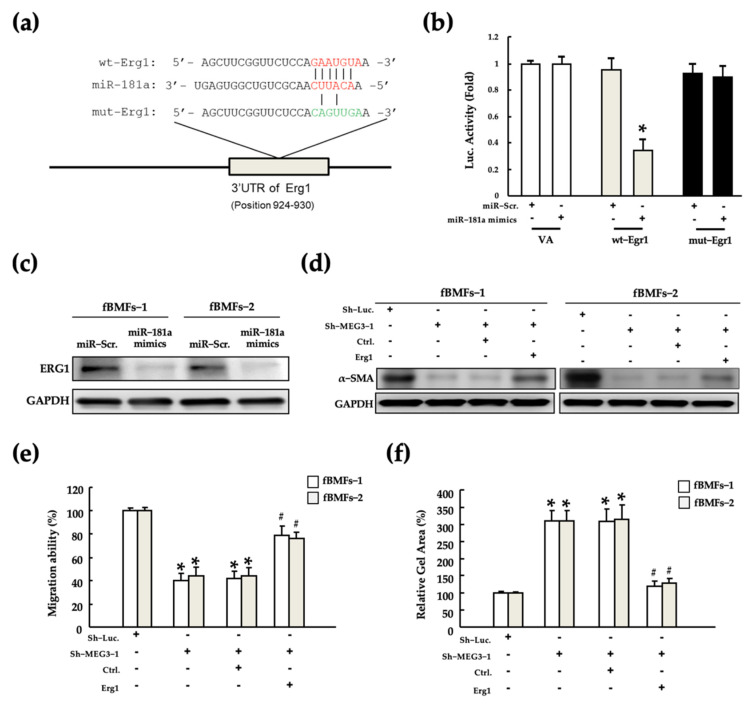
MEG3 promoted the myofibroblast activities of patient-derived fBMFs by regulating the miR-181a/Egr1 axis. (**a**) The putative base pairing sequence of wild-type (wt) and mutated (mut)-Egr1 between with miR-181a. (**b**) Relative luciferase activity of each combination was examined in two fBMFs * *p* < 0.05 compared with miR-Scr; non-target vector (VA). (**c**) Changes in α-SMA expression were examined in fBMFs after transfection with miR-Scr or miR-181a mimics. Changes in (**d**) α-SMA expression and (**e**) migration and (**f**) gel contraction abilities were examined in two fBMFs after co-transfection with or without Sh-Luc, Sh-MEG3, Ctrl. and Egr1, respectively. * *p* < 0.05 compared with Sh-Luc; # *p* < 0.05 compared with co-transfection with Sh-MEG3 and Ctrl.

The miR-181a family mediates renal fibrosis development [29,48] and cancer progression [49,50] by targeting Egr1. Moreover, some studies revealed that miR-181a and Egr1 are involved in various oral diseases [51,52,53]. However, whether the miR-181a/Egr1 axis is established and as functions in fBMFs remain unknown. Thus, to explore the presence of the miR-181a/Egr1 axis in fBMFs, we established a putative miR-181a binding site on the 3’UTR sequence of Egr1, which was verified by performing a luciferase reporter assay (Figure 6a,b, respectively). Luciferase reporter activity was not changed in fBMFs with mut-Egr1 after co-transfection with miR-181a mimics but was significantly decreased in fBMFs with wt-Egr1. Consistent with the luciferase reporter activity, Egr1 protein expression was significantly decreased after transfection with miR-181a mimics (Figure 6c). These results indicate that miR-181a inhibited the expression of Egr1 by directly interacting with Egr1 at the translational level. Furthermore, to explore whether the MEG3/miR-181a/Egr1 axis functionally exists in fBMFs, we next manipulated the expression of MEG3 and Egr1, and evaluated the changes in myofibroblast activities. Silencing of MEG3 significantly reduced α-SMA expression and migration and gel contraction abilities; however, this inhibitory effect was partially rescued by the overexpression of Egr1 (Figure 6d–f). These results demonstrate that MEG3 acts as a competing endogenous RNA (ceRNA) for Egr1 by functioning as a miR-181a sponge. These findings demonstrate the pro-fibrotic role of the MEG3/miR-181a/Egr1 axis in OSF and indicate that the MEG3/miR-181a/Egr1 axis might be a target for fucoidan treatment.

## 3. Discussion

Fucoidan exerts several therapeutic effects, including immunomodulatory [30,31], anti-cancer [54], and anti-fibrosis [36,55]. Because OSF is regarded as a precancerous disorder, the effect of fucoidan on the tissue fibrosis caused by cancer therapy caught our attention. Fucoidan ameliorated radiation-induced lung fibrosis by decreasing inflammatory cytokine expression [36], and attenuated radioiodine-induced salivary gland injury and fibrosis [55]. To our knowledge, the therapeutic effects of fucoidan on oral fibrosis remain unclear. Our results demonstrated the anti-fibrotic of fucoidan on OSF by suppressing the myofibroblast activities of patient-derived fBMFs. It should be noted that we only ensured that fucoidan had no cytotoxicity on BMFs (Figure 1). However, normal fibroblasts also share many characteristics with myofibroblasts but at less levels, such as lower expression of α-SMA and lower secretion of type 1 collagen (Col-1) and TGF-β1 [56]. It remains unclear whether fucoidan affects the functions of BMFs, which may influence the safety assessment of fucoidan for developing a therapeutic drug. Although knowledge of the effect of fucoidan on normal fibroblasts is still lacking, some previous studies indicate that fucoidan has no adverse effect on normal tissue compared with fibrotic tissue. Li et al. demonstrated that fucoidan exhibited protective effects against the carbon tetrachloride (CCI4)/bile duct ligation (BDL)-induced liver fibrosis [57]. Compared with the sham group, no significant change was observed in Col-1, α-SMA, matrix metallopeptidase (MMP)-9 and tissue inhibitor of metalloproteinase 1 (TIMP-1) expression, and TGF-β signaling pathway activity in healthy mice liver tissue following the administration of fucoidan. Yu et al. demonstrated that fucoidan inhibited radiation-induced pulmonary fibrosis by reducing the expression of inflammatory cytokines [36]. Administration of fucoidan to healthy mice had no significant effect on the Col-1 deposition in lung tissue and the expression of pro-inflammatory cytokines in pleural fluid compared with the sham group. Moreover, no significant change was observed in releasing Col-1α from NIH-3T3 fibroblasts when cultured with the pleural fluid derived from fucoidan-treated mice. Zhang et al. reported that fucoidan diminished the hyperoxia-induced lung injury in newborn rats by suppressing the myofibroblasts [58]. Their results revealed no significant change in the α-SMA and Col-1 expression between mouse primary lung fibroblasts isolated from the sham and fucoidan treatment group. Taken together, the aforementioned results suggest that fucoidan may target abnormally activated fibroblasts (e.g., myofibroblasts) in fibrosis conditions but not normal fibroblasts. In addition, numerous studies have reported that fucoidan treatment promotes the healing efficiency of skin wounds. For instance, Song et al. demonstrated that fucoidan enhanced fibroblast proliferation and increased the expression of proliferating cell nuclear antigen (PCNA), p63 and α-integrin in the human in vitro skin equivalent [59]. Park et al. revealed that treatment with low molecular weight fucoidan (LMWF) on rat full-thickness dermal excision wound showed a similar healing effect to those of treatment with Madecasol Care™ (a commercial nature source) [60]. Interestingly, LMWF-stimulated upregulation of TGF-β1 at the injury site was considered a beneficial effect on improving wound repair [60], but fucoidan inhibited tissue fibrosis by suppressing the TGF-β signaling pathway [57,61,62]. Therefore, the effect of fucoidan on fibroblasts may depend on the type of tissue, pathogenic condition, and the response in the crosstalk of microenvironmental factors, such as cytokines and growth factors.

Our work demonstrated that fucoidan suppressed the myofibroblast activities of fBMFs by downregulating the lncRNA MEG3 expression. Moreover, we noted that MEG3 was significantly correlated with OSF and was overexpressed in clinical OSF tissue compared with their corresponding normal tissues. Although MEG3 has been reported as the anti-fibrotic and anti-cancer lncRNA, some studies can support our findings. For instance, treatment with fucoidan significantly downregulated MEG3 expression in hepatocarcinoma cells [34]. Furthermore, the inhibition of MEG3 prevented cardiac fibrosis and diastolic dysfunction by reducing MMP-2 expression [61]. The overexpression of MEG3 in the lung epithelial cells of the idiopathic pulmonary fibrosis (IPF) model disrupted their epithelial differentiation and promoted the EMT process [62]. Oral epithelial cells dysfunction caused by areca nut chewing is a major pathogenic mechanism underlying oral fibrosis and cancer [16]. Therefore, determining the clinical significance and underlying mechanism of MEG3 in OSF progression is essential. In the present study, we observed that MEG3 acted as a ceRNA to sponge miR-181a and thus prevented the miR-181a-mediated inhibition of Egr1 expression in fBMFs. Moreover, we noted the pro-fibrotic effects of the MEG3/miR-181a/Egr1 molecular axis, including increases in TGF-β1 secretion and myofibroblast activities. Consistent with our finding, Zha et al. reported that the MEG3/miR-181a/Egr1/TLR4 axis enhances diabetic nephropathy inflammation and fibrosis [29]. However, it is hard to assess the impact of fucoidan-mediated MEG3 on BMFs because we only examined the role of MEG3 in fBMFs. A recent study demonstrated that selenium suppressed the homocysteine-induced fibrotic activation in cardiac fibroblasts by inhibiting the MEG3 [63]. Notably, the expression of MEG3, alpha-SMA, Col-1, and Col-3 showed no significant difference between fucoidan-treated or non-treated healthy mouse primary cardiac fibroblasts. This evidence hints that the fucoidan-mediated MEG3 axis may not affect the normal fBMFs. However, the phenotype and function of the fibroblast are heterogeneous in fibrosis progression [64,65]. Thus, the identification of MEG3 levels in BMFs and fBMFs by the RNA sequencing technique and the examination of the effect of fucoidan on high or low MEG3 expressing fibroblasts subpopulation in the future study are necessary for the verification of the anti-fibrotic role of fucoidan in OSF progression.

MiR-181a is involved in various biological events, including immune cell aging and cancer development and progression [66,67,68]. Moreover, miR-181a exerts the pro-fibrotic effect in various types of tissues. For instance, miR-181a acts as a pro-fibrotic miRNA by promoting renal fibrosis through the targeting of fibroblast growth factor 1 (FGF1) [69]. Overexpression of miR-181a in hepatocytes caused a change in the EMT phenotype similar to that observed after TGF-β treatment [70]. The increase in miR-181a expression was induced by TGF-β, thus downregulating ALR to activate hepatic stellate cells [69]. However, our study revealed the anti-fibrotic function of miR-181a in fBMFs. We noted that the overexpression of miR-181a inhibited myofibroblast activities in fBMFs by targeting Egr1, which plays a critical role in arecoline-induced oral fibrosis [51,71]. Wu et al. indicated that Egr1 is essential for myofibroblast transdifferentiation and the wound healing process [72]. An in vitro study demonstrated that the lncRNA COX prevents aberrant epidural fibrosis by targeting Egr1 [73]. TGF-β is a major factor driving fibrosis progression. Egr1 enhanced the TGF-β signaling by targeting its promoter and thus promoted high glucose-induced proliferation and ECM production in rat glomerular mesangial cells [74]. Egr1 is involved in regulating chronic hypoxia-induced renal fibrosis by targeting the PKC/ERK pathway [75]. Furthermore, miR-181a exhibits an anti-fibrotic effect by targeting Egr1. For instance, Zhang et al. reported that miRNA-181 directly targets Egr1, resulting in the downregulation of pro-fibrotic markers in the renal fibrosis model [48]. Xu et al. reported that the downregulation of miR-181a-5p induced by a high glucose concentration rescued Egr1 expression and increased pro-fibrotic gene expression in human renal cells [76]. Furthermore, epigallocatechin-3-gallate (EGCG), a catechin present in green tea, exerted the anti-fibrotic effect on arecoline-induced fibrosis by inhibiting Egr1. Hsieh et al. demonstrated that Egr1 is overexpressed in the OSF tissue. A low dose of arecoline stimulated the upregulation of Egr1, and Egr1 expression was suppressed after treatment with EGCG in fBMFs [71]. Arffe et al. indicated that miR-181a was among the most upregulated miRNAs in HepG2 cells after treatment with EGCG [75].

Although this study did not determine mechanisms through which Egr1 regulates oral fibrosis, some studies have suggested that its pro-fibrotic effect can be attributed to its involvement in the modulation of inflammatory responses. Zha et al. demonstrated that the MEG3/miR-181a/Egr1 axis mediates the inflammatory response and fibrosis in diabetic nephropathy by regulating Toll-like receptors (TLR) 4 [29]. Similarly, Wu et al. reported that the inhibition of the Egr1/TLR4/mTOR axis reduced the expression of fibrosis and inflammatory cytokines in high glucose-stimulated rat renal cells [77]. Constant exposure to a poor oral environment, including dental plaque, prolonged drug administration and areca nuts chewing, markedly increases the risk of chronic and uncontrollable inflammation in the oral tissue [78]. TLRs are crucial for inflammation, proliferation, and ECM production in oral cells [78]. Inflammation stimulates TLRs and activates their downstream targeting genes, such as NF-κB, IL-6, and numerous pro-inflammatory cytokines. We observed that the silencing of MEG3 reduced the IL-6 secretion in fBMFs (Figure 3). Moreover, NF-κB is involved in the inflammation-induced fibrosis progression in various types of tissues [79,80,81]. The lncRNA MALAT1 promotes liver fibrosis by targeting the miR-181a/TLR4/NF-κB axis [82]. Taken together, the aforementioned results support our finding that MEG3 promotes OSF progression by sponging miR-181a to increase Egr1 expression. In addition, we demonstrated that fucoidan alleviates myofibroblast activities by targeting the MEG3/miR-181a/Egr1 axis, and this MEG3 network might depend on the type of tissues or pathogenic mechanism (areca nut chewing, diabetes, and inflammation).

The limitation of the present study is the lack of in vivo evidence. Since the establishment of OSF animal models is time-consuming and there are associated technical difficulties (treated with aqueous areca nut extracts for 300 to 600 days) [83], we examined the effect of fucoidan on BMFs and fBMFs in priority. Therefore, the next step in our future study will establish an OSF-animal model induced by treatment with arecoline and investigate the therapeutic effect of fucoidan and targeting MEG under the early and advanced stages of OSF progression.

**Figure 7:**
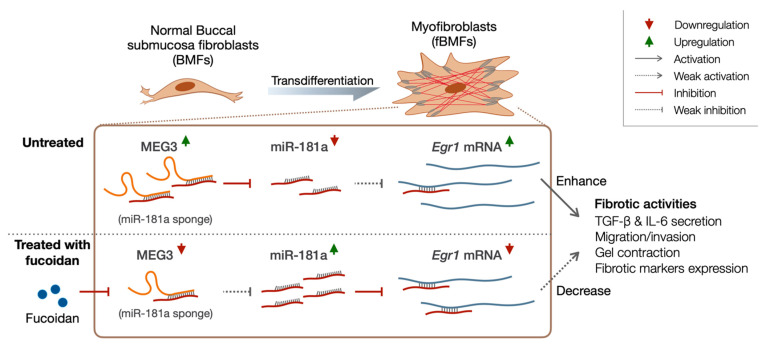
Schematic diagram of the role of the MEG3/miR-181a/Egr1 molecular axis in regulation of myofibroblast features in OSF progression. MEG3 acted as a miRNA sponge to decoy away miR-181a from Egr1 mRNA, thus leading to the upregulation of Egr1 expression by restoring its translational process. Overexpression of Egr1 promoted the expression of the fibrotic genes, ultimately leading to the activation of myofibroblasts. Fucoidan exerted an anti-fibrotic effect on patient-derived fBMFs via suppressing the expression of MEG3 expression, thus resulting in the disruption of the MEG3/miR-181a/Egr1 axis. Red, solid line with vertical head indicates the inhibiting effect; gray, dotted line with arrowhead indicates the weakened increasing effect; red arrowhead indicates the downregulation.

A schematic diagram in Figure 7 summarizes novel therapeutic mechanisms through which fucoidan can treat OSF in the present study. Our findings demonstrated that the administration of fucoidan downregulated MEG3, consequently alleviating the activation of myofibroblasts. We noted a significantly positive correlation between MEG3 overexpression and OSF progression. The possible pro-fibrotic mechanism of MEG3 in fBMFs might be acting as a ceRNA to Egr1 by functioning as an miR-181a sponge, thus restoring the expression of Egr1. In summary, our study indicated that fucoidan have the potential to prevent the exacerbation of OSF by mediating the MEG3/miR-181a/Egr1 molecular axis.

## 4. Materials and Methods

### 4.1. Cell Culture and Reagents

All procedures were approved by the Institutional Review Board of Taipei Medical University, Taipei, Taiwan (TMU-JIRB No: N202103142). All the tissue specimens of healthy individuals and patients with OSF were collected from the Department of Dentistry, Chung Shan Medical University Hospital, after written informed consent was obtained. Buccal mucosal fibroblasts (BMFs) were isolated from the normal human buccal oral mucosa tissue, which was obtained during surgical removal of the impacted mandibular third molars. (BMF-1, male, 67 years old; BMF-2, male, 43 years old; male). Fibrotic buccal mucosa fibroblasts (fBMFs) were isolated from the fibrotic buccal oral mucosa with OSF patients (fBMF-1, male, 54 years old; fBMF-2, male, 53 years old). The detailed tissue collection and primary cell culture methods were described in our previous study [84]. In brief, normal and OSF tissues were cut into 1–2 mm pieces and cultured in a 25T-flask with Dulbecco’s modified Eagle’s medium (DMEM) containing 10% fetal bovine serum (FBS). Primary BMFs or fBMFs were migrated from the tissue margin and were passaged routinely at 90% confluence. The third and eighth passage cells were used in the subsequent experiments. Unless otherwise specified, fucoidan and all reagents were purchased from Sigma-Aldrich (St. Louis, MO, USA).

### 4.2. Cell Viability Assay

All the cells were seeded into 96-well-plates at a density of 1 × 10^4^ cells/well and incubated at 37 °C for 48 h. The old medium was replaced with the fresh medium containing 0.1% dimethyl sulfoxide (DMSO; as control) or designated concentration (10, 20, or 40 μg/mL) of fucoidan. After 24 h of incubation, PrestoBlue viability reagent (Thermo Fisher Scientific, Carlsbad, CA, USA) was directly added into each well, and the plate was incubated for another 2 h. Colorimetric absorbance of each well at 570 and 600 nm was measured using the Synergy H4 Hybrid microplate reader (Agilent BioTek Instruments, Winooski, VT, USA).

### 4.3. Cell Migration Assay

The cell migration ability was evaluated by performing Transwell and wound-healing assays. For the Transwell assay, 1 × 10^5^ cells suspended in a serum-free medium were added into the Transwell insert chamber with a membrane pore size of 8 μm (Corning, Acton, MA, USA). Then, the culture medium containing 10% FBS was added into the lower chamber to induce chemotaxis. After incubation for 24 h, the migrated cells were fixed with 100% methanol for 30 min and stained with 0.1% crystal violet for another 30 min. The number of the migrated cells was counted in five random images by using an inverted microscope. For the wound-healing assay, the cells were seeded into 12-well plates. After 90% cells reached confluence, a straight wound area was directly scraped by using a sterile 200-μL pipette tip. Subsequently, the cells were incubated with a serum-free medium for 48 h. Cell movement into the wound area center was photographed at 0 and 48 h by using an inverted microscope [84].

### 4.4. Collagen Gel Contraction Assay

The cells were suspended in a 0.5 mL of 2 mg/mL collagen gel solution and added into a 24-well plate, followed by incubation at 37 °C for 2 h. After gel polymerization, 0.5 mL of the culture medium was added into each well and subsequently scraped a sterilized flat with a stainless-steel spatula around the well-wall to release the gel disc. The culture plate was constantly incubated for 48 h. The change in collagen gel size (contraction index) was photographed by using an inverted microscope and measured using ImageJ software (NIH) [84].

### 4.5. Enzyme-Linked Immunosorbent Assay

Enzyme-linked immunosorbent assay (ELISA) was used to detect the secretion of TGF-β and IL-6 from BMFs and fBMFs. When the 90% cell confluence was reached, cells were exposed to a serum-free medium for 24 h of incubation. Then, the cultured supernatant was collected and centrifuged to remove dead cells. TGF-β1 and IL-6 secretion in the cultured medium were measured using the human TGF-β1 ELISA kit and IL-6 ELISA kit (Invitrogen, Carlsbad, CA, USA), respectively, in accordance with the manufacturer’s instructions.

### 4.6. Western Blotting

The preparation of fBMFs before the total protein extraction can be described as follows. For the fucoidan treatment experiments, fBMFs were seeded into a 6-well plate at a density of 5 × 10^5^ cells/well and incubated at 37 °C for 24 h. Then, cells were exposed to the fresh cultured medium with or without fucoidan. After another 24 h of incubation, cells were harvested for protein extraction. For the transfection experiment, fBMFs after transfection (see Section 4.9) for 72 h were transferred into a 6-well plate at a density of 5 × 10^5^ cells/well and incubated at 37 °C for 24 h. Then, cells of equal confluence were harvested for protein extraction. The harvested cells were lysed with a RIPA lysis buffer containing 1× protease inhibitor cocktail (Invitrogen Life Technologies, Carlsbad, CA, USA), and the protein concentration was quantitated using the Bradford protein assay (Bio-Rad, Santa Rosa, CA, USA). Whole-cell lysates (containing 40 μg protein) were separated by 10% SDS-PAGE electrophoresis and transferred onto a nitrocellulose membrane (Millipore, Billerica, MA, USA). The membranes were blocked with 5% bovine serum albumin (BSA) for 1 h and then incubated at 4 °C with the following primary antibodies: anti-α-SMA (Cat. MA5-11547), anti-COL1A1 (Cat. PA1-26204), anti-Smad (Cat. 436500), anti-pSmad2 (Cat. 44-244G) and anti-GAPDH (Cat. MA5-15738). After 16 h of incubation, the membranes were washed with Tris-buffered saline with 0.1% Tween-20 (TBST) buffer for 5 min three times and then incubated with the corresponding secondary antibodies for 2 h at room temperature. The immunoreactive bands were developed using an ECL-plus chemiluminescence substrate (Millipore, Billerica, MA, USA) and subsequently captured using the LAS-1000 plus luminescent image analyzer (GE Healthcare, Piscataway, NJ, USA). All the antibodies were purchased from ThermoFisher Scientific (Carlsbad, CA, USA) [84].

### 4.7. RNA Isolation and Sequencing

High-throughput RNA sequencing was performed to screen for the putative targets to identify the differentially expressed genes in fBMFs after fucoidan treatment. Total RNAs was isolated from three individual fBMFs treated or non-treated with fucoidan using a Trizol reagent. The quality of RNA extracted from each sample was ensured by the manufacturer of Genomics Inc. After RNA-seq library construction, discrepancies in the transcriptome within cells were detected and analyzed using the FPKM method (fragments per kb of transcript per million mapped reads) by using HiSeq2500 (Illumina, San Diego, CA, USA) [85].

### 4.8. Real-Time Quantitative Polymerase Chain Reaction

Total RNA isolated from the tissues and cells by following the aforementioned procedure described in the above subsection. cDNAs was synthesized from total RNA in accordance with the manufacturer’s instructions by using the Superscript III first-strand synthesis system (Invitrogen Life Technologies, Carlsbad, CA, USA). Real-time quantitative polymerase chain reaction (qRT-PCR) analysis was performed using the ABI StepOne™ Real-Time PCR System (Applied Biosystems, Foster City, CA, USA). The sequences of specific PCR primers were listed as follows: MEG3: 5′–GCATTAA–GCCCTGACCTTTG–3′ (forward) and 5′–TCCAGTTTGCTAGCAGGTGA–3′ (reversed); and GAPDH: 5′–TTAAAAGCAGCCCTGGTGAC–3′ and 5′–CTCTGCTCCTCCTGTTCGAC–3′.

### 4.9. Lentiviral-Mediated Knockdown or Overexpression

For MEG3 silencing, lentiviral particles expressing the non-target control or shRNA against MEG3 were purchased from Sigma-Aldrich (St. Louis, MO, USA) and used for transduction in accordance with the manufacturer’s instructions. For Egr1 overexpression, Egr1 cDNA was cloned into pLV-EF1a-MCS-IRES-Puro (BioSettia, CA, USA). Lentiviruses were produced through the co-transfection of the plasmid DNA mixture with the lentivector plus helper plasmids (VSVG and Gag-Pol) into 293T cells by using Lipofectamine 2000 reagent (LF2000, Invitrogen, Carlsbad, CA, USA). The transfection efficiency was confirmed by conducting qRT-PCR. The miR-181a mimic, inhibitor, and miR-181a scramble were purchased from ThermoFisher Scientific (Carlsbad, CA, USA). For inhibition and overexpression of miR-181a, the miR-181a inhibitor and mimic were transfected by using Lipofectamine 2000 reagent, respectively.

### 4.10. Dual-Luciferase Reporter Assay

The sequence of MEG3 or Egr1-3′-UTR containing the predicted wild-type or mutant binding sites of miR-181a was cloned and inserted into a pmirGLO plasmid (Promega, Madison, WI, USA) to generate the pmirGLO luciferase reporter plasmids in accordance with the manufacturer’s instructions. The luciferase reporter plasmids containing MEG3 or Egr1-3′-UTR were then co-transfected into cells with the miR-181a mimic, inhibitor, or scramble by using Lipofectamine 2000 reagent. The relative luciferase activity was measured in accordance with the manufacturer’s instructions [84].

### 4.11. RNA Immunoprecipitation Assay

The RNA Immunoprecipitation (RIP) assay was performed using the Magna RIP kit (Millipore, Billerica, MA, USA) in accordance with the manufacturer’s instructions. For immunoprecipitation, whole-cell lysates were incubated with RIP immunoprecipitation buffer containing the Ago2 antibody (Abcam, Burlingame, CA, USA) and NC IgG (Abcam, Cambridge, MA, USA)-conjugated magnetic beads. After 2 h of incubation, precipitated RNA was purified to detect the expression of MEG3 and miR-181a through qRT-PCR [84].

### 4.12. Statistical Analysis

All statistical analyses was performed using IBM SPSS Statistics (SPSS 27 for Windows). We performed Student’s *t*-test and one-way analysis of variance (ANOVA) with post hoc Dunnet’s and Tukey’s tests. A *p*-value of < 0.05 was considered statistically significant. All data are presented as mean ± standard deviation (SD) from at least three independent experiments.

## Data Availability

Data is contained within the article.
